# Structure-Based Design and Antigenic Validation of Respiratory Syncytial Virus G Immunogens

**DOI:** 10.1128/jvi.02201-21

**Published:** 2022-03-10

**Authors:** Ana M. Nuñez Castrejon, Sara M. O’Rourke, Lawrence M. Kauvar, Rebecca M. DuBois

**Affiliations:** a Department of Microbiology and Environmental Toxicology, University of California Santa Cruz, Santa Cruz, California, USA; b Department of Biomolecular Engineering, University of California Santa Cruz, Santa Cruz, California, USA; c Trellis Bioscience, Inc., Redwood City, California, USA; University of Kentucky College of Medicine

**Keywords:** monoclonal antibodies, respiratory syncytial virus, structure-activity relationships, vaccines

## Abstract

Respiratory syncytial virus (RSV) is a leading cause of severe lower respiratory tract disease of children, the elderly, and immunocompromised individuals. Currently, there are no FDA-approved RSV vaccines. The RSV G glycoprotein is used for viral attachment to host cells and impairment of host immunity by interacting with the human chemokine receptor CX3CR1. Antibodies that disrupt this interaction are protective against infection and disease. Nevertheless, development of an RSV G vaccine antigen has been hindered by its low immunogenicity and safety concerns. A previous study described three engineered RSV G proteins containing single-point mutations that induce higher levels of IgG antibodies and have improved safety profiles compared to wild-type RSV G (H. C. Bergeron, J. Murray, A. M. Nuñez Castrejon, et al., Viruses 13:352, 2021, https://doi.org/10.3390/v13020352). However, it is unclear if the mutations affect RSV G protein folding and display of its conformational epitopes. In this study, we show that the RSV G S177Q protein retains high-affinity binding to protective human and mouse monoclonal antibodies and has equal reactivity as wild-type RSV G protein to human reference immunoglobulin to RSV. Additionally, we determined the high-resolution crystal structure of RSV G S177Q protein in complex with the anti-RSV G antibody 3G12, further validating its antigenic structure. These studies show for the first time that an engineered RSV G protein with increased immunogenicity and safety retains conformational epitopes to high-affinity protective antibodies, supporting its further development as an RSV vaccine immunogen.

**IMPORTANCE** Respiratory syncytial virus (RSV) causes severe lower respiratory diseases of children, the elderly, and immunocompromised populations. There currently are no FDA-approved RSV vaccines. Most vaccine development efforts have focused on the RSV F protein, and the field has generally overlooked the receptor-binding antigen RSV G due to its poor immunogenicity and safety concerns. However, single-point mutant RSV G proteins have been previously identified that have increased immunogenicity and safety. In this study, we investigate the antibody reactivities of three known RSV G mutant proteins. We show that one mutant RSV G protein retains high-affinity binding to protective monoclonal antibodies, is equally recognized by anti-RSV antibodies in human sera, and forms the same three-dimensional structure as the wild-type RSV G protein. Our study validates the structure-guided design of the RSV G protein as an RSV vaccine antigen.

## INTRODUCTION

Respiratory syncytial virus (RSV) is the leading cause of severe lower respiratory disease in children and infants, causing approximately 33 million cases and 118,000 deaths globally every year (1–3). RSV is also a major contributor of respiratory disease in the elderly and immunocompromised populations, causing approximately 10,000 deaths annually (4–8). The only FDA-approved prophylaxis is the monoclonal antibody (MAb) palivizumab (Synagis), which reduces hospitalizations due to RSV infection but does not prevent infection (9–11). There currently is no FDA-approved vaccine available to protect against RSV infection.

RSV contains two immunogenic envelope glycoproteins that elicit neutralizing antibodies: RSV F and RSV G (12). The membrane fusion glycoprotein, RSV F, exists in a prefusion form (RSV pre-F) that undergoes a conformational change to post-F in order to cause membrane fusion (12). RSV F is the target of most serum neutralizing antibodies, and thus it has been the focus of most monoclonal antibody and vaccine developmental strategies (13, 14). However, RSV F has known variability and antibody escape potential, and escape from an anti-F monoclonal antibody suptavumab was responsible for its failure in phase 3 trials (15, 16).

The attachment glycoprotein, RSV G, has important roles in RSV infection and in impairment of host immunity. RSV G on the virus surface promotes virus attachment to human airway epithelial cells by interacting with the human chemokine receptor CX3CR1 (17–20). RSV G impairs host immunity through diverse mechanisms. RSV G dampens the type I antiviral interferon (IFN) responses in airway epithelial cells and dendritic cells, limiting host innate defenses against RSV infection (21). Furthermore, RSV G protein induces a Th2-biased cytokine response in CD4^+^ T cells (22, 23) and downregulates Th1-mediated immune responses, at least in part by modulating neonatal regulatory B cells (24). Notably, RSV G is produced as both a membrane-bound form, for incorporation into new virion particles, as well as a secreted form (RSV sG) due to a conserved second start codon at Met 48 (25–27). RSV sG has been shown to compete with CX3CL1 for binding to CX3CR1, modulating signaling and trafficking to the lungs by CX3CR1^+^ killer T cells and NK cells, which are needed for clearance of RSV-infected cells (20, 28–30). RSV sG has also been shown to modulate the responses of Fc-expressing immune cells, limiting their ability to clear RSV-antibody complexes and RSV-infected cells (31).

Despite RSV G’s role in infection and disease, it has been mostly overlooked as a vaccine target due to its overall sequence variability, highly glycosylated mucin-like domains, and safety concerns (described below) (12, 32, 33). In addition, RSV G is less immunogenic than RSV F, with RSV G eliciting approximately 2 to 10% of human serum neutralizing antibodies (34, 35). RSV G does, however, contain a nonglycosylated region that is nearly invariant across strains, termed the central conserved domain (CCD), which interacts with CX3CR1 and is the target of broadly neutralizing antibodies (36–44). Structural studies have revealed that the RSV G CCD contains conformational epitopes where these protective antibodies bind (45–47).

Anti-RSV G antibodies that target the CCD have been shown to be protective *in vivo* in mouse and cotton rat models of RSV infection. The anti-RSV G mouse monoclonal antibodies (MAbs) 131-2G, 5A6, and 3A5 and the human MAbs 3D3 and 3G12 reduce lung viral loads and weight loss in mouse models (36–44, 48). Anti-RSV G MAbs also reduce disease symptoms, including pulmonary inflammation, airway resistance, and mucus production in mouse models (36–38, 40–42, 44, 48). Notably, the bivalent antigen binding fragment [F(ab′)_2_] of 131-2G reduces pulmonary cell infiltration, mucin production, weight loss, and airway dysfunction without reducing lung viral titers in mice, revealing that anti-RSV G antibody protection from disease symptoms is not due solely to reduced viral loads (36, 41). Moreover, F(ab′)_2_ 131-2G shifted the antibody response toward a Th1 response, with higher IgG2a antibody titers in immunized mice, and lowered the percentage of interleukin-4 (IL-4)-positive T cells (48). In cotton rats, anti-RSV G antibody CB017.5 reduced viral loads, inflammation, and pathology in both treatment and prophylactic models (47).

Anti-RSV G antibodies that target the CCD are also protective in human models *in vitro*. MAbs 131-2G, CB002.5, and CB017.5, as well as others, directly neutralize RSV infection in primary human airway and bronchial epithelial cells (17, 19, 47, 49). One study developed an *in vitro* model of RSV infection. Peripheral blood mononuclear cells (PBMCs) were cocultured in the top chamber of a permeable membrane Transwell insert with RSV-infected human airway epithelial cells in the bottom chamber (50). The addition of F(ab′)_2_ 131-2G to this *in vitro* system decreased virus replication and increased the levels of alpha interferon (IFN-α) and tumor necrosis factor alpha (TNF-α) produced by plasmacytoid dendritic cells and monocytes within PBMCs (50). A separate study demonstrated anti-RSV G MAbs can induce the destruction of RSV-infected Hep-2 cells by PBMCs and phagocytosis by human blood neutrophils (49). Finally, in a study in human infants and young children, lower clinical disease severity upon natural RSV infection was correlated with higher maternal immunoglobulin G antibodies against RSV G and RSV pre-F (*P* = 0.028 and 0.038, respectively), despite the >30-fold lower abundance of antibodies against RSV G compared to RSV pre-F (35).

Despite the abundance of evidence that anti-RSV G antibodies are protective *in vivo*, poor immunogenicity and safety concerns have hindered the development of RSV G as a vaccine antigen. In a 1960s’ clinical trial of formalin-inactivated RSV (FI-RSV), vaccine recipients experienced enhanced respiratory disease (ERD) upon natural RSV infection (51–55). Subsequent studies have identified several factors that may have contributed to ERD, including lung eosinophilia, a Th2-biased immune response, and low-avidity antibody responses leading to immune complex deposition in the lungs (56). RSV G as a vaccine antigen—either as a recombinant protein or vaccinia virus expressing RSV G—recapitulates some aspects of ERD in the mouse model: mainly eosinophilia and increased Th2 cytokines/chemokines following RSV challenge (51–55). Furthermore, immunization with sG increases the levels of the Th2 cytokines IL-5, which activates eosinophils, and IL-13 produced in the lungs of immunized mice (54, 57). However, subsequent studies revealed that the immune responses from FI-RSV and RSV G antigen alone are distinct (23, 58), and there are contradictions in the literature as to whether immunization with FI-RSV from RSV lacking the G protein reduces ERD following RSV challenge (30, 59). Nevertheless, the ability of RSV G antigen to prime for eosinophilia and a Th2-biased response has hindered its development as a vaccine antigen.

One strategy to improve the safety and immunogenicity of the RSV G antigen has involved mutagenesis to inactivate the detrimental activities tied to the CCD. In one approach, mutagenesis of the CX3C motif to CX4C (insertion of an alanine before cysteine 186) was used to develop a live attenuated RSV vaccine (RSV CX4C). Evaluation of RSV CX4C in an *in vitro* model of RSV infection of human airway epithelial (HAE) cells incubated with human PMBCs revealed that RSV CX4C induced higher levels of type I and III interferons compared to wild-type (WT) RSV (50). Additionally, RSV CX4C is less effective at infecting HAE cells, which may decrease its pathogenicity (18, 60). Evaluation of RSV CX4C compared to wild-type RSV *in vivo* showed reduced lung inflammatory cell infiltration, mucus production, and airway resistance upon RSV challenge in mice (18, 50, 60, 61). Furthermore, RSV CX4C induced higher serum neutralizing antibody titers compared to wild-type RSV (61). Cotton rats, which are more permissible to RSV disease than mice, infected with RSV CX4C had less interstitial inflammation and alveolitis compared to rats infected with wild-type RSV (60).

In another approach, single-point mutagenesis of a different CCD residue, serine 177, which is conserved as a small amino acid glycine or serine, was compared to the CX4C mutant in an RSV G subunit vaccine (62). In this study, mice immunized with recombinant RSV G proteins containing wild-type or CX4C, S177Q, or S177R mutant sequences were evaluated for antibody responses and markers of disease. RSV G S177Q and S177R proteins induced higher levels of anti-RSV IgG antibodies in immunized mice before and after RSV challenge compared to mice immunized with RSV G wild-type or CX4C proteins, as assessed by enzyme-linked immunosorbent assay (ELISA). Moreover, elicited antibodies were shown to block RSV G-CX3CR1 interaction, which is a known protective functional activity of anti-RSV G antibodies (described above). Additionally, mice immunized with RSV G S177Q protein had reduced bronchoalveolar lavage cell influx into the lungs.

Despite the promise of RSV G engineering to improve immunogenicity and overcome safety concerns, it has been unclear if the mutations affect antigen folding and display of RSV G conformational epitopes. This is important because structural distortion of RSV G protein could prevent binding of high-affinity antibodies and reduce vaccine efficacy. In this study, we compare RSV G wild-type and CX4C, S177Q, and S177R mutant proteins for their binding affinities to anti-RSV G MAbs 3G12, 3D3, 2D10, and 131-2G. Additionally, we compare RSV G wild-type and mutant proteins for binding to human reference immunoglobulin to RSV. Finally, we present the structure of antibody 3G12 bound to RSV G CCD S177Q to evaluate at the atomic level the mutant CCD folding and display of the 3G12 conformational epitope. These studies validate the structure and antigenicity of the RSV G S177Q mutant and support its further development as an RSV vaccine immunogen.

## RESULTS

### Design of RSV G^ecto^ WT and mutant proteins.

**FIG 1:**
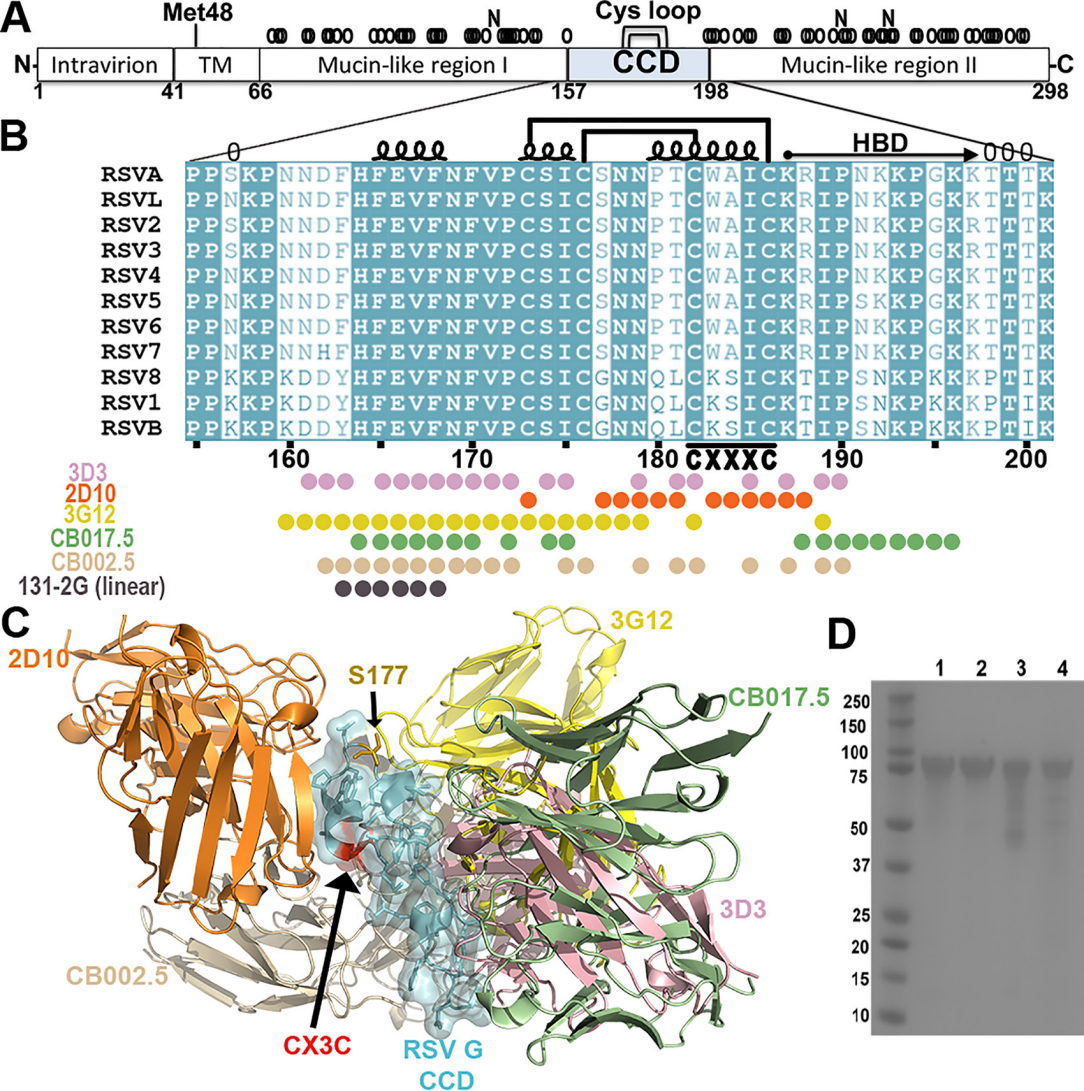
Anti-RSV G monoclonal antibody interactions with the RSV G central conserved domain (CCD). (A) A schematic of RSV G protein of RSV strain A2 showing the transmembrane domain (TM), the alternate initiation site at methionine 48 that initiates the production of soluble RSV G protein, mucin-like regions I and II with predicted O- and N-linked glycans (“0” and “N,” respectively) above, the CCD (amino acids [aa] 157 to 198), and the 4-cysteine loop (Cys loop) within the CCD. (B) Sequence alignment of the RSV G CCD from indicated strains. Conserved amino acids are highlighted in cyan. Secondary structure and disulfide bonds are represented by loops and brackets, respectively. The heparin binding domain (HBD) is labeled. The CX3C motif is shown at amino acids 182 to 186. Conformational epitope amino acids for anti-RSV G monoclonal antibodies 3D3 (pink), 2D10 (orange), 3G12 (yellow), CB017.5 (green), and CB002.5 (wheat) and the linear epitope amino acids for 131-2G (gray) are indicated. It is unknown if antibody 131-2G has a larger or conformational epitope. (C) RSV G CCD (side view) shown in cyan, with S177 highlighted in gold and the CX3C motif highlighted in red. Variable domains of monoclonal antibodies 2D10 (orange; PDB code 5WN9), 3G12 (yellow; PDB code 6UVO), CB017.5 (green; PDB code 6BLH), 3D3 (pink; PDB code 5WNA), and CB002.5 (wheat; PDB code 6BLI) when bound to overlaid RSV G CCD structures are displayed. (D) Coomassie-stained SDS-PAGE gel of RSV G^ecto^ WT and mutant proteins at ∼90 kDa. Lane 1, WT; lane 2, S177Q mutant; lane 3, S177R mutant; lane 4, CX4C mutant.

Recombinant RSV G ectodomain (G^ecto^) wild-type (WT) and mutant (CX4C, S177Q, and S177R) proteins (residues 64 to 298) were designed, expressed, and secreted in mammalian cells and purified from the cell medium as described previously (62). Briefly, RSV G^ecto^ CX4C was engineered by insertion of an alanine into the CX3C motif before cysteine 186 (50) (Fig. 1A). RSV G^ecto^ S177 mutants were designed by investigating all known structures of the RSV G CCD bound to human antibodies 3G12, 3D3, 2D10, CB002.5, and CB017.5 to identify CCD amino acid side chains that do not contribute to the conformational epitopes (45–47) (Fig. 1A and B). Serine 177, which is conserved as a small amino acid glycine or serine, was mutated to a larger amino acid, glutamine, or arginine (S177Q and S177R, respectively), to potentially sterically disrupt interactions with CX3CR1 (Fig. 1C). Analysis of purified RSV G proteins confirmed purity (Fig. 1D). However, it is unclear from these studies if the mutant RSV G proteins fold correctly or display known conformational epitopes.

### RSV G^ecto^ S177 mutant proteins retain high-affinity binding to anti-RSV G MAbs.

To determine whether recombinant RSV G^ecto^ mutant proteins retain conformational epitopes for anti-RSV G MAbs, binding analyses were conducted using Octet biolayer interferometry. Anti-human or anti-mouse IgG Fc biosensors were loaded with anti-RSV G human MAbs (3G12, 2D10, and 3D3) or mouse MAb 131-2G, respectively. Biosensors were submerged into 1:2 serial dilutions of RSV G^ecto^ WT or mutant proteins to measure on-rates and then submerged into buffer to measure off-rates (Table 1). The binding constant (equilibrium dissociation constant [*K_D_*]) for each antibody-antigen interaction was then calculated (Table 1).

**TABLE 1:** Binding affinity constant (*K_D_*), on-rates (*k_a_*), off-rates (*k_d_*), *R*^2^, and χ^2^ of RSV G^ecto^ WT and mutant proteins to anti-RSV G MAbs

Sample	MAb	*K_D_* (pM)	*k_a_* (10^5^ M^−1^s^−1^)	*k_d_* (10^−5^ s^−1^)	*R* ^2^	χ^2^
RSV G^ecto^ WT	3G12	260	2.52	6.55	0.9992	0.4802
		262	1.98	5.19	0.9993	0.4809
RSV G^ecto^ S177Q	3G12	2,280	2.32	52.8	0.9974	0.6908
		1,880	2.38	44.7	0.9975	0.5193
RSV G^ecto^ S177R	3G12	4,340	1.34	58.1	0.9949	1.4869
		4,600	1.46	67.2	0.9965	0.8916
RSV G ^ecto^ CX4C	3G12	27,700	0.399	110	0.9751	0.5311
		28,000	0.367	103	0.9681	0.6455
RSV G^ecto^ WT	2D10	<1	3.70	<0.01	0.998	0.8776
		<1	3.83	<0.01	0.9975	1.0426
RSV G^ecto^ S177Q	2D10	605	3.71	22.5	0.9985	0.5392
		485	3.1	15	0.9959	1.4183
RSV G^ecto^ S177R	2D10	<1	3.18	<0.01	0.9983	0.6609
		<1	2.53	<0.01	0.9962	1.1652
RSV G^ecto^ CX4C	2D10	65,100	0.249	162	0.9848	0.6959
		93,300	0.213	199	0.9839	0.5709
RSV G^ecto^ WT	3D3	<1	4.26	<0.01	0.9988	0.5743
		<1	4.31	<0.01	0.9983	0.5431
RSV G^ecto^ S177Q	3D3	538	3.69	19.9	0.9974	0.4947
		264	4.14	10.9	0.9982	0.2435
RSV G^ecto^ S177R	3D3	422	2.47	10.4	0.9972	0.6466
		570	2.65	15.1	0.9972	0.6919
RSV G^ecto^ CX4C	3D3	6,080	0.531	32.3	0.9946	0.6276
		6,230	0.479	29.8	0.9893	0.7662
RSV G^ecto^ WT	131-2G	1,900	2.87	54.4	0.9891	0.1445
		3,200	2.89	92.4	0.9609	0.4062
RSV G^ecto^ S177Q	131-2G	3,480	3.38	118	0.9928	0.102
		6,540	2.3	151	0.9875	0.1745
RSV G^ecto^ S177R	131-2G	8,600	0.481	41.4	0.9933	0.1485
		2,360	2.48	58.6	0.9853	0.4089
RSV G^ecto^ CX4C	131-2G	13,200	0.477	62.8	0.9591	0.2116
		19,600	0.496	97	0.9813	0.1074

RSV G^ecto^ WT protein bound to MAbs 3D3, 3G12, and 131-2G with a low-picomolar, high-picomolar, and low-nanomolar *K_D_*, respectively, consistent with previous binding studies (39, 46, 49) (Table 1). We also determined for the first time that RSV G^ecto^ WT protein binds to MAb 2D10 with a low-picomolar *K_D_*, similar to the known highest-affinity anti-RSV G Mab, 3D3. RSV G^ecto^ S177Q and S177R mutant proteins had the same or modestly reduced affinity for all four MAbs compared to RSV G^ecto^ WT protein. In the cases of reduced binding, affinities were still maintained at high-picomolar or low-nanomolar *K_D_*s. Together, these data indicate that the S177Q or S177R mutations do not disturb the epitopes required to bind diverse anti-RSV G MAbs with high affinity.

In contrast, RSV G^ecto^ CX4C protein had reduced binding affinities for all four anti-RSV G MAbs (Table 1). While binding affinity was only modestly reduced for MAb 131-2G, affinities for MAbs 3D3 and 3G12 were reduced by approximately 100-fold, and affinity to MAb 2D10 was reduced by approximately 10,000-fold. These data reveal that epitopes of the RSV G^ecto^ CX4C mutant protein are significantly disrupted by the mutation and have reduced affinities for diverse anti-RSV G MAbs.

### Human reference immune globulin to RSV equally recognizes RSV G^ecto^ WT and S177Q mutant proteins.

To determine whether RSV G^ecto^ WT and mutant proteins could be recognized by anti-RSV antibodies in human sera, enzyme-linked immunosorbent assays (ELISAs) were conducted using human reference immune globulin to RSV (human anti-RSV Ig), which has been shown to contain RSV neutralizing antibodies (63, 64). ELISA plates were coated with recombinant RSV G^ecto^ WT and mutant proteins, and reactivity to serially diluted human anti-RSV Ig was evaluated.

**FIG 2:**
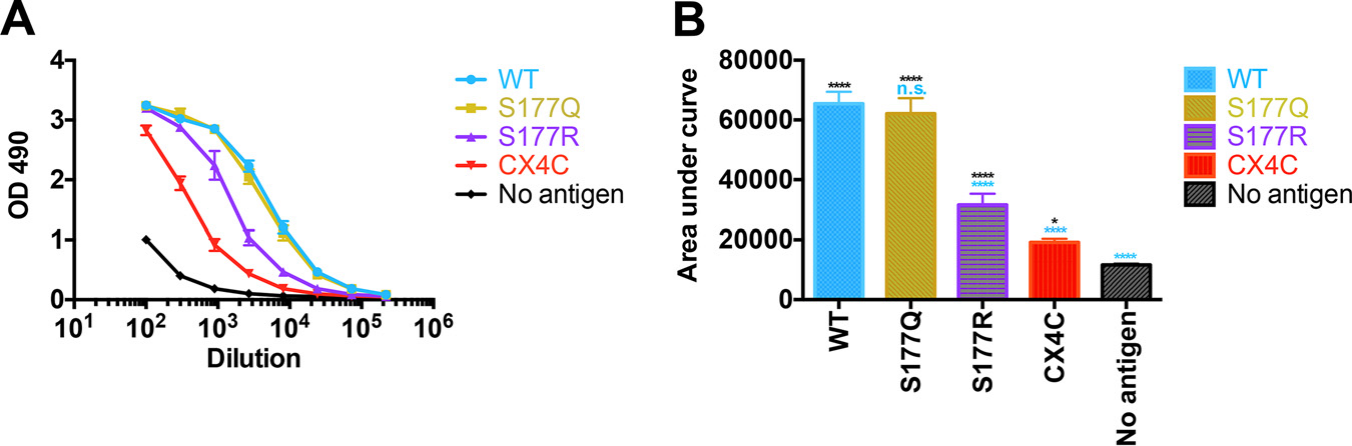
Human reference immunoglobulin to RSV binding to RSV G^ecto^ WT and mutant proteins. (A) Dilution series ELISA to evaluate the human reference IgG reactivity to recombinant RSV G^ecto^ WT and mutant proteins. Samples were evaluated in biological quadruplicates, and error bars represent 1 standard deviation (SD) from the mean. A curve is shown for RSV G^ecto^ WT (cyan) and S177Q (gold), S177R (purple), and CX4C (red) mutant proteins, as well as a negative control with no antigen (black). (B) The area under the curve for each sample was calculated in Prism. Error bars represent the mean and SD. Data were analyzed by a one-way analysis of variance (ANOVA): n.s., not significant (*P* ≥ 0.05); *, *P* = 0.01 to 0.05; ****, *P* < 0.0001.

ELISA signals and area under the curve (AUC) calculations are shown in Fig. 2. RSV G^ecto^ WT and S177Q mutant proteins had indistinguishable reactivities, suggesting that the immunodominant epitopes in human anti-RSV Ig are not significantly affected by the S177Q mutation. In contrast, RSV G^ecto^ S177R and CX4C mutant proteins had reduced reactivities compared to RSV G^ecto^ WT. RSV G^ecto^ CX4C had the lowest reactivity of the three RSV G^ecto^ mutant proteins, consistent with MAb binding studies; however, it had some detectable reactivity over the “no-antigen” control (*P* = 0.01 to 0.05). The data indicate that the RSV G^ecto^ S177Q mutant protein displays immunodominant epitopes for human anti-RSV Ig.

### Fab 3G12-RSV G CCD S177Q structure.

**FIG 3:**
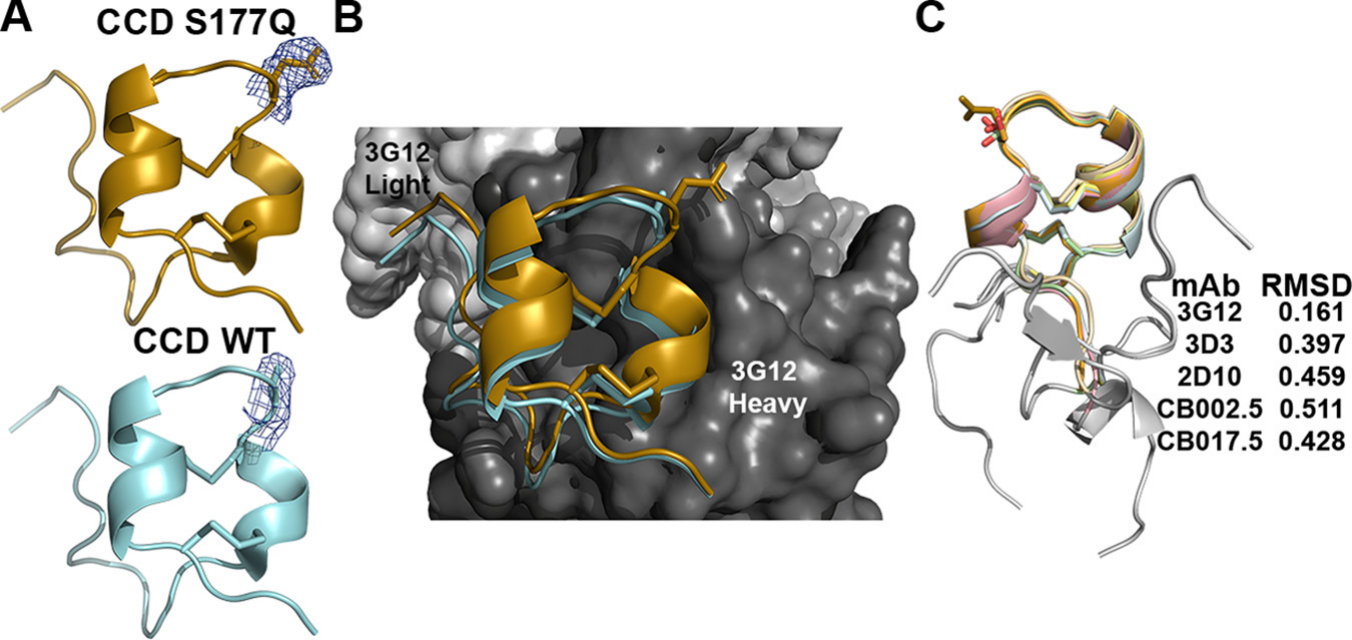
Crystal structure of Fab 3G12 in complex with RSV G CCD S177Q at a 3.1-Å resolution. (A) Comparison of RSV G CCD WT (cyan; PDB code 6UVO) and S177Q (gold; PDB code 7T8W) when bound to Fab 3G12. Electron density maps, contoured at 1.0 sigma within 1.8 Å of amino acid 177, are shown. (B) Overlay of the structures of RSV G CCD WT (cyan) and RSV G CCD S177Q (gold) bound to Fab 3G12. (C) Overlay of RSV G CCD WT bound to anti-RSV G antibodies 3G12 (yellow; PDB code 6UVO), 3D3 (pink; PDB code 5WNA), and 2D10 (orange; PDB code 5WN9) and CB002.5 (wheat; PDB code 6BLI), CB017.5 (green; PDB code 6BLH), and RSV G CCD S177Q bound to 3G12 Fab (gold; PDB code 7T8W). Flexible amino acids are shown in gray. RMSD values were determined using PyMOL.

To further show at the atomic level that the S177Q mutation does not disrupt CCD folding and display of conformational epitopes, we used X-ray crystallography to solve the structure of RSV G CCD S177Q bound to 3G12 antibody (Fig. 3 and Table 2). Recombinant RSV G CCD residues 157 to 197 containing the S177Q mutation were copurified with the fragments of antigen binding (Fab) 3G12. The complex was crystallized, and the structure was solved to 3.1-Å resolution. The structure reveals that the 3G12 conformational epitope is displayed by the RSV G CCD containing the S177Q mutant, consistent with MAb 3G12 binding studies showing high-affinity binding. The electron density maps show extended density for the mutant glutamine side chain compared to the density of the wild-type serine side chain at RSV G residue 177 (Fig. 3A). Comparison of the RSV G CCD wild-type and S177Q mutant structures shows that they are nearly identical, with a root mean square deviation (RMSD) of 0.16 Å, and their contacts with antibody 3G12 are largely conserved (Fig. 3B). Notably, although residue 177 is in the middle of a loop constrained by the four cysteines in two disulfide bonds, the S177Q mutation does not prevent the formation of these disulfide bonds, which are important for the display of all known conformational epitopes. Indeed, structural comparison of RSV G CCD S177Q with wild-type RSV G CCDs bound to different antibodies reveals that the central disulfide-bonded regions of the CCDs (residues 169 to 187) are nearly identical (Fig. 3C). Overall, this structure reveals that the RSV G CCD containing the S177Q mutation is structurally intact and displays conformational epitopes.

**TABLE 2:** Data collection and refinement statistics for Fab 3G12-RSV G CCD S177Q complex

Parameter	PDB code 7T8W^*a*^
Data collection statistics	
Space group	P 31 2 1
Unit cell dimensions	
*a*, *b*, *c* (Å)	139.39, 139.39, 98.05
α, β, γ (°)	90, 90, 120
Resolution (Å)	98.05–3.10 (3.31–3.10)
* R*_sym_ or *R*_merge_	0.705 (3.614)
* I*/σ〈*I*〉	5.8 (1.8)
Completeness (%)	100 (100)
Redundancy	19.1 (18.7)
CC_1/2_	0.974 (0.567)

Refinement statistics	
No. of reflections	20,274 (1,983)
Resolution (Å)	76.106–3.1 (3.211–3.1)
* R*_work_/*R*_free_	0.2369/0.2500
No. of atoms:	
Protein	3,598
Ligands	0
Water	0
B factors	
Protein	53.35
Ligands	0
Water	0
RMSD	
Bond length (Å)	0.014
Bond angle (°)	2.03
Ramachandran statistics (%)	
Favored	96.13
Allowed	3.87
Outliers	0

a Data from one crystal were used for each structure determination. Values in parentheses are for the highest-resolution shell.

## DISCUSSION

The value of a structure-based approach for the improvement of vaccine antigens has become widely appreciated (65). Here, we investigated human antibody reactivities to three RSV G mutant proteins (CX4C, S177Q, and S177R), which were previously shown to have increased immunogenicity and safety *in vivo* compared to wild-type RSV G protein (62). The CX4C mutation had been designed to disrupt the CX3C motif, which is associated with detrimental RSV G activities. However, the CX4C mutation had been designed before antibody-RSV G complex structures and conformational epitopes had been elucidated. The S177 mutations had been designed with these structures in mind to minimize disruption of conformational epitopes.

Affinity binding studies for MAbs against the RSV G S177 mutant proteins reveal that their conformational epitopes are largely intact (Table 1). MAb binding affinity constants remained in the picomolar or low-nanomolar range. Interestingly, in all cases of reduced binding, such as the 17-fold reduction in binding of MAb 3G12 to RSV G S177R protein, analyses of binding kinetics reveal that the on-rates were the same as for binding to wild-type RSV G, but the off-rates were slightly faster (Table 1). As the S177 side chain is only 3.5 to 4.0 Å away from MAb 3G12 CDR3 residues (Fig. 3B), it is likely that the size or charge of the S177R mutation caused some repulsion and affected off-rates. On the other hand, the S177 side chain is >10 Å away from MAb 3D3 residues, yet S177 mutations modestly reduced MAb 3D3 binding. This suggests that CCD mutations may affect MAb binding by mechanisms other than direct repulsion—perhaps by restricting CCD flexibility and entropy.

In contrast to studies with RSV G S177 mutant proteins, MAb binding affinity studies with the RSV G CX4C mutant protein reveal that its conformational epitopes are significantly disrupted (Table 1). Binding of MAb 2D10 to RSV G CX4C protein was severely reduced (10,000-fold), which is not surprising given that the CX3C motif forms ∼40% of the 2D10 epitope. On the other hand, binding to MAb 3G12 was reduced by approximately 100-fold, despite the CX3C motif forming only 0.01% of the 3G12 epitope, suggesting that the CX4C mutation induces global changes to the CCD structure beyond just the CX3C motif. Consistent with this, a 6-fold reduction in binding to MAb 131-2G was observed, despite the CX4C mutation being 17 amino acids downstream of the 131-2G linear epitope (Fig. 1B), suggesting that the CX4C mutation broadly distorts the CCD structure and/or MAb 131-2G may have a conformational epitope. Notably, analyses of binding kinetics reveal that both on-rates and off-rates are significantly affected by the CX4C mutation (Table 1).

We further evaluated the RSV G mutant proteins in ELISA studies with human reference immune globulin to RSV. Consistent with MAb binding studies, ELISA studies reveal that the RSV G CX4C mutant protein has significantly reduced binding to human anti-RSV antibodies compared to wild-type RSV G. Interestingly, we observed a difference between the two RSV G S177 mutant proteins: whereas the RSV G S177R protein has reduced binding compared to wild-type RSV G, the RSV G S177Q protein has equal binding to human anti-RSV antibodies compared to wild-type RSV G. We note that a generally understood distinction between binding affinity studies with human MAbs and ELISAs with human anti-RSV antibodies is that MAb studies evaluate individual epitopes, whereas ELISAs with human serum antibodies are biased in favor of immunodominant epitopes. These data suggest that the S177Q mutation has little or no effect on the immunodominant epitopes of RSV G protein.

Finally, we validated the structure of RSV G S177Q protein by solving the high-resolution structure of its CCD bound to antibody 3G12. The structure confirms the structural integrity of CCD with the S177Q mutation and is consistent with human MAb and human anti-RSV antibody binding studies.

Altogether, these studies validate for the first time that RSV G immunogens can be engineered for increased immunogenicity and safety while retaining conformational epitopes to high-affinity protective antibodies. In particular, our studies, in combination with those of Bergeron et al. (62), identify the RSV G S177Q mutant protein as a promising RSV vaccine immunogen. The RSV G S177Q mutant protein could be studied further as a subunit vaccine or integrated into other candidate RSV vaccine platforms, including subunit, virus-like particles, live attenuated RSV, virus-vectored, and mRNA vaccines (14). We note that several RSV vaccine candidates targeting the RSV pre-F antigen are currently in phase 3 clinical trials, with some preliminary efficacy data from phase 2 clinical trials suggesting approximately 70% efficacy against symptomatic RSV infection, an improvement over previous RSV vaccine candidates targeting RSV post-F but with potential for additional improvement. Mutational escape from the MAbs against the F protein has already proven to be clinically significant, and widespread use of F protein vaccines or MAbs given prophylactically runs the risk of generating escape mutants. Notably, none of these candidates includes an RSV G immunogen, meaning that they will not elicit the broadly protective anti-RSV G antibodies that block RSV engagement of the human CX3CR1 receptor, which is key for infection of human airway epithelial cells and for RSV dampening of host immune responses. Thus, addition of engineered RSV G immunogens to RSV pre-F vaccine candidates may increase both efficacy and universality while reducing escape potential.

## MATERIALS AND METHODS

### Expression and purification of wild-type and mutant RSV G^ecto^ proteins.

A synthetic gene encoding RSV (strain A2) G protein ectodomain (RSV G^ecto^) amino acids 64 to 298 (UniProtKB entry P03423), in frame with an N-terminal tissue plasminogen activator (TPA) or CD5 signal sequence and tandem C-terminal 6 histidine and Twin-Strep purification tags, was cloned into cytomegalovirus (CMV) promoter-driven expression plasmid pCF or a derivative of pcDNA3.1 (66). Mutations in the RSV G gene were introduced by Phusion site-directed mutagenesis and verified by Sanger sequencing. The CX4C mutant contains an additional alanine within the CX3C motif before the second cysteine (C^186^) to encode CWAIAC (CX4C) instead of the wild-type sequence CWAIC (CX3C). Serine 177 was mutated to glutamine or arginine for the S177Q and S177R mutants, respectively. Recombinant RSV G^ecto^ proteins were expressed by transient transfection in CHO-S cells using electroporation (Maxcyte STX) and purified from the medium by StrepTrap affinity chromatography. All proteins were concentrated to 1 mg/mL and dialyzed into phosphate-buffered saline (PBS). Purity was verified by SDS-PAGE (Fig. 1D).

### Anti-RSV G MAbs 3D3, 2D10, 3G12, and 131-2G.

Synthetic genes encoding the heavy-chain or light-chain variable regions of human MAb 2D10 were cloned by Gibson assembly into the pCMVR VRC01 antibody vectors for light and heavy chains obtained from the AIDS Reagent Program, in place of the variable regions of antibody VRC01, a human anti-HIV antibody (IgG1) targeting the gp120 protein (67). The plasmids were verified by DNA sequencing. Recombinant MAb 2D10 was expressed by transient transfection in CHO-S cells and purified from the medium by immobilized protein A affinity chromatography. Purity was verified by SDS-PAGE. Recombinant MAbs 3D3 and 3G12 were produced as described previously and obtained from Trellis Bioscience (39). Mouse MAb 131-2G was purchased from EMD Millipore (MAB858-2).

### Binding affinity analyses.

An Octet RED384 biolayer interferometry instrument was used to determine binding affinities of wild-type and mutant RSV G^ecto^ proteins to anti-RSV G MAbs. Anti-human IgG Fc capture AHC biosensors (for MAbs 3D3, 2D10, and 3G12) or anti-mouse IgG Fc capture AMC biosensors (for MAb 131-2G) were submerged in Octet buffer (PBS [pH 7.4], 0.05% Tween 20, 1% bovine serum albumin [BSA]) for 60 s. The sensors were loaded for 120 s with 1 µg/mL of human or mouse anti-RSV G MAb and then submerged into Octet buffer for 120 s. Association of MAbs with wild-type and mutant RSV G^ecto^ was measured for 300 s, and dissociation in Octet buffer was measured for 600 s. RSV G^ecto^ proteins started at 40 nM and were 2-fold serially diluted to 0.63 nM to measure binding to anti-RSV G MAbs. Higher starting concentrations were required to be able to fit curves for RSV G^ecto^ CX4C protein. A global association 1:1 model was used to fit at least three curves to determine the on- and off-rates to calculate the equilibrium dissociation constant (*K_D_*). All binding assays were performed in duplicate.

### ELISA with anti-RSV human immune globulin.

Flat-bottom, high-binding Costar 3590 ELISA plates were coated with wild-type and mutant RSV G^ecto^ proteins at 1 µg/mL in PBS at 4°C overnight. The plates were washed three times with wash buffer (PBS [pH 7.4], 0.1% Tween 20). Plates were blocked overnight at 4°C with blocking buffer (PBS [pH 7.4], 5% dry milk, 1% BSA). The blocking buffer was decanted, and human reference immune globulin to respiratory syncytial virus (CBER RSV lot 1; BEI Resources no. NR-21973) was added starting at a 1:100 dilution in blocking buffer and serially diluted 3-fold for 1 h at 37°C. The plates were washed three times with wash buffer. Goat anti-human IgG Fc secondary antibody conjugated to horseradish peroxidase (HRP) (Invitrogen no. A18817) was added at a 1:3,000 dilution for 1 h at 37°C. The plates were washed two times with wash buffer. Plates were developed for 10 min with phosphate citrate buffer, 12.5 µL of 30% hydrogen peroxide, and two *o*-phenylenediamine dihydrochloride (OPD) tablets. The reaction was stopped with 2 N sulfuric acid, and the plates were measured at the optical density at 490 nm (OD_490_) using a Molecular Devices Spectramax plate reader. Human anti-RSV G MAb 3G12 was used as a positive control, starting at 1 µg/mL and serially diluted 3-fold. No antigen was used as a negative control. Curves were graphed in GraphPad Prism and are the average of four replicates.

### Expression and purification of RSV G CCD S177Q.

A synthetic gene encoding RSV (strain A2) G CCD amino acids 157 to 197 (UniProtKB entry P03423) was cloned into pRSFDuet-1 with an N-terminal methionine and a C-terminal 6-histidine tag. The S177Q mutation was introduced by Phusion site-directed mutagenesis and verified by Sanger sequencing. Recombinant RSV G CCD S177Q was expressed in T7Express Escherichia coli overnight at 18°C. Cells were lysed by ultrasonication in wash buffer (20 mM Tris [pH 8], 25 mM imidazole, 150 mM NaCl) with 0.1 mM MgCl_2_, protease inhibitors, and benzonase. RSV G CCD S177Q was purified from clarified E. coli lysates by affinity chromatography using a HisTrap FF crude column and washed with wash buffer containing 6 M urea. Protein was eluted in wash buffer containing 500 mM imidazole.

### Fab preparation of 3G12.

The Fab fragments of human anti-RSV G MAb 3G12 were produced using the Pierce Fab preparation kit (Thermo Scientific 44985). Briefly, MAb 3G12 was incubated with immobilized papain in PBS for 5 h at 37°C. Fc fragments were removed using immobilized protein A. Fab purity was verified by SDS-PAGE.

### Purification of Fab 3G12-RSV G CCD S177Q complex structure.

To generate the Fab 3G12-RSV G CCD S177Q complex, Fab 3G12 was incubated with a 4-molar excess of RSV G CCD S177Q on ice for 30 min. A Superdex 75 size exclusion chromatography column was used to purify the complex and eluted in 10 mM Tris-HCl (pH 8) and 150 mM NaCl. The complex was concentrated to 8.26 mg/mL. Crystals were grown by hanging drop vapor diffusion in a well solution of 1.8 M ammonium sulfate and 100 mM sodium acetate trihydrate (pH 4.4) at room temperature. Crystals were transferred to a cryoprotectant solution of 2 M ammonium sulfate, 100 mM sodium acetate trihydrate (pH 4.4), 25% glycerol, and 18% of a 1:1:1 ratio of 100% ethylene glycol - dimethyl sulfoxide - glycerol (EDG) and flash frozen in liquid N_2_. Diffraction data were collected at the Advanced Light Source on beamline 5.0.1. Data were processed using DIALS and scaled using Aimless (within CCP4). Phaser was used for molecular replacement, and Phenix Refinement and Coot were used for structure refinement.

### Data availability.

Coordinates and structure factors for the Fab 3G12-RSV G CCD S177Q complex structure have been deposited in the Protein Data Bank under accession code 7T8W. All other data have been made available in this article.
